# *Bacillus amyloliquefaciens* CU33 Fermented Feather–Soybean Meal Product Improves the Crude Protein Digestibility, Diarrhea Status, and Growth Performance of Goat Kids

**DOI:** 10.3390/ani14192809

**Published:** 2024-09-29

**Authors:** Tsung-Yu Lee, Yueh-Sheng Lee, Chean-Ping Wu, Kun-Wei Chan, Kuo-Lung Chen

**Affiliations:** 1 Animal Nutrition Division, Taiwan Livestock Research Institute, Ministry of Agriculture, Tainan 712009, Taiwan; johntyli@mail.tlri.gov.tw; 2 Ph.D. Program of Agriculture Science, National Chiayi University, Chiayi 600355, Taiwan; lopqkojhog@gmail.com; 3 Department of Animal Science, National Chiayi University, Chiayi 600355, Taiwan; wcp@mail.ncyu.edu.tw; 4 Department of Veterinary Medicine, National Chiayi University, Chiayi 600355, Taiwan; kwchan@mail.ncyu.edu.tw

**Keywords:** *Bacillus amyloliquefaciens* CU33, digestibility, feather meal–soybean meal product, goat kid, growth performance

## Abstract

**Simple Summary:**

Providing high-quality feeds is required to maintain goat kids’ health and improve their growth performance during their early growth phase, such as milk protein, fish meal, or extruded feed. The present study demonstrates that supplementation with 2% fermented feather meal–soybean meal product in the diet can promote the growth of goat kids by improving crude protein digestibility, diarrhea status, and immunity: therefore, it can completely replace the fish meal as a starter. These findings provide potential for practices of nutrition and immunity in goat kids, benefiting the livestock industry.

**Abstract:**

This study investigates the effects of replacing fish meal with fermented feather meal–soybean meal product (FFSMP) from *Bacillus amyloliquefaciens* CU33 in the starter on growth performance, relative health performance, and digestibility of Alpine goat kids. In trial 1, thirty-two Alpine goat kids (male) were randomly assigned to dietary supplementation of 2% feather meal–soybean meal mix (FSM), 2% fish meal, or replacing fish meal with 50% or 100% FFSMP (FFSMP-50 or FFSMP-100) in starter (n = 8). In trial 2, sixteen goat kids were selected after trial 1 and used in this digestion trial which began from 12 weeks old. The treatments were the same as in trial 1 (n = 4). In trial 1, the growth performance of the FFSMP groups was better than the FSM group at 0 to 10 weeks (*p* < 0.05). The fecal consistency index of the FFSMP-100 group was better than the FSM group at 0 to 5 weeks and 5 to 10 weeks. In trial 2, the crude protein (CP) digestibility of the FSM group decreased (*p* < 0.05). In conclusion, dietary supplementation with 2% FFSMP in goat kids’ diets can improve the growth performance, the CP digestibility, and diarrhea status, and it can completely replace the fish meal in starter diets.

## 1. Introduction

Goats in Taiwan are typically mated from August to October, resulting in kids being born from January to March. After birth, goat kids receive colostrum and are subsequently raised on milk replacer and trough feeding starts at three days of age until weaning, which occurs at 8 to 10 weeks. Diarrhea often occurs in goat kids with sudden drops in the ambient temperature or changes in diet, which can delay weaning until 12 weeks of age. During this early growth phase, the digestion of goat kids resembles that of monogastric animals, as their rumen has not yet fully developed. Providing high-quality feed is essential for maintaining their health and enhancing their growth performance later on [1,2]. Generally, a good-quality starter from milk protein, fish meal, or extruded feed can significantly improve the growth performance of young ruminants [3,4]. Currently, the prices of fish meal and milk powder prices have increased due to the COVID-19 pandemic, which has led to higher transportation costs. Therefore, the search for alternative animal protein sources is the primary goal at present.

Probiotics benefit the growth and immunity of young animals, particularly *Bacillus* spp., which can improve immune performance and promote the development of the rumen [5,6]. Hong and Wu mixed feather and soybean meals in a 9:11 ratio and then inoculated them with *Bacillus* spp. for 2 days [2]. Supplementing this product for 5% of the feed improved weight gain and feed conversion ratio in goat kids aged 6 to 8 weeks. In our previous research, we mixed feather and soybean meals in a 1:1 ratio and inoculated them with *Bacillus* spp., protease, and keratin-decomposing enzymes for 2 days. This was followed by 5 days of anaerobic fermentation, during which we inoculated with *Bacillus* coagulans L12, known for its strong acid-producing ability, to create two-stage fermentation products. Feeding these fermentation products as a complete replacement for the high-quality fish meal in growing and finishing pigs promoted their growth and alleviated diarrhea problems [7,8].

Fermentation using only complex *Bacillus* strains resulted in unstable quality. The second-stage fermentation, however, is time-consuming and increases overall production costs. Therefore, our research team screened a single strain, *B. amyloliquefaciens* CU33 (CU33), which exhibits a strong ability to hydrolyze feather and soybean proteins. We then inoculated CU33 into a mixture of feather meal and soybean meal (1:1) with an adjusted moisture content of 50–60% for 2 days of aerobic fermentation, resulting in a fermented feather meal–soybean meal product (FFSMP). Adding 5% FFSMP to the broiler diet significantly improved the growth performance of broilers and enhanced the duodenal villus height to crypt depth ratio, allowing for a complete replacement of high-quality fish meals [9].

However, the FFSMP from CU33 has not been used in the diets of goat kids, and its effect on their health is unclear. Therefore, this study aimed to investigate the physiological characterizations of the FFSMP from CU33 and its potential to replace high-quality fish meals in goat kids’ starter diets. The study assessed its effects on growth performance, health performance, and digestibility.

## 2. Materials and Methods

### 2.1. Fermented Feather Meal–Soybean Meal Product Preparation

Mixed feather meal and soybean meal at a ratio of 1:1 was supplied as fermentation substrate. The moisture of substrate was adjusted to 45% and sterilized at 121 °C, 1.21 kg/cm^2^ for 20 min and cooled down to 45 °C. The fermentation substrate was inoculated with CU33 (10^7^ cfu/g), and aerobic fermentation was conducted at 37 °C for 48 hr. The fermented product was dried in an oven at 55 °C. The moisture of the FFSMP was below 12%, and three batches were produced for the current study.

### 2.2. The Physiochemical Characterizations and Nutrient Composition of FFSMP

The pH of FFSMP was measured by a portable pH meter (digital pH meter, Goodly, Taiwan). The FFSMP was serially diluted in 0.85% NaCl and incubated on tryptic soy agar (TSA, HIMEDIA^®^, Mumbai, MH, India) at 37 °C for 24 h. In colony counting for *Bacillus*-like colonies are expressed as colony-forming units per gram. The γ-PGA of FFSMP was measured by the method of Goto and Kunioka [10]. The viscosity of FFSMP was evaluated on a 5-point scale where 1 is the best score (viscosity: 1 = not sticky; odor: 1 = most acceptable) and 5 is the worst score (viscosity: 5 = very sticky; odor: 5 = most unacceptable). The protease activity of FFSMP refers to the methods of Moreno-Hernández et al. [11]. The keratinolytic activity was assayed, followed by Huang et al. [8]. The surfactin yield of FFSMP was measured by the method of Lee et al. [12]. Proximate analysis of FFSMP followed the description of AOAC [13] to analyze the moisture (method 930.15), crude protein (CP) (method 990.03), calcium (Ca) (method 927.02), and phosphorus (P) (935.59). The gross energy (GE) was measured with an adiabatic bomb calorimeter (model 356, Parr Instrument Company, Moline, IL, USA). The amino acid analyses of FFSMP followed the description of Yeh et al. [14].

### 2.3. Animal Management and Experimental Design

In trial 1, thirty-two 14-d-old Alpine goat kids (male) were randomly assigned to four dietary supplementation groups of 2% feather meal–soybean meal mix (FSM), 2% fish meal, or replacing fish meal with 50% or 100% FFSMP (FFSMP-50 or FFSMP-100) as starter. Each treatment had eight replicates. The experimental period was 10 weeks, and the milk replacer (Victoria Whole Milk Powder, Victoria, Australia) (CP 24.5%, fat 26.3%, lactose 40.3%, mineral 5.8%, and moisture 3.1%) was prepared in 136 g DM/L at 38 °C. During the first week of the trial period, the goat kids were fed milk replacer three times a day at a volume of 450 mL. From the second to the tenth week, the feeding frequency was reduced to twice a day, with a volume of 700 mL. The goat kids were completely weaned by the end of the tenth week of the trial. Feed (Table 1) and water were provided ad libitum throughout the experimental period. The kids were individually raised in stainless steel cage (width 70 cm × height 70 cm × depth 80 cm) to facilitate the calculation of feed intake and observation of feces.

In trial 2, sixteen Alpine goat kids (male, 12-wk of age) were selected from the end of trial 1 and were randomly assigned to dietary supplementation of 2% FSM, 2% fish meal, FFSMP-50 or FFSMP-100. Each treatment had four replicates. The experimental period was 7 days. Feed (Table 1) and water were provided ad libitum throughout the experimental period. All the procedures used in this experiment were approved by the Institutional Animal Care and Use Committee of National Chiayi University (111039).

### 2.4. Proximate Feed Analysis

Proximate feed analyses were carried out as described previously to analyze the moisture, CP, Ca, and P. Neutral detergent fiber (NDF) and acid detergent fiber (ADF) were measured, following the description of Van Soest et al. [15].

### 2.5. Growth Performance

In trial 1, body weight (BW), total feed intake (FI), and starter intake were recorded at weeks 0, 5, and 10. Weight gain (WG) and feed conversion ratio (FCR) were calculated throughout the experiment.

### 2.6. General Health Performance

In trial 1, the number of diarrhea stools, the number of days of diarrhea, the rate of diarrhea, and the number of therapeutic treatments in goat kids were recorded every day. The incidence of various diseases was estimated from the number of antibiotic treatments assigned by farm personnel or the veterinarian against digestive, respiratory, or other diseases such as joint or umbilicus infections. The general health score (GHS) was measured following the description of Timmerman et al. [16] with some modifications. The incidence of diarrhea and therapeutic treatments for digestive, respiratory, or other diseases were weighted differently in the following formula: GHS per animal = 35 − 1 × total number of diarrheic days (irrespective of its nature)—2 × the number of individual therapeutic treatments for digestive diseases—3 × the number of individual therapeutic treatments for respiratory diseases—2 × the number of individual therapeutic treatments for infections other than digestive or respiratory—2 × the number of antibiotic treatments on a herd basis. The weighting factor of each abnormality was based on its assumed impact on health.

The fecal appearance and consistency were recorded, scored, and assigned four values according to the fecal consistency index (FCI) modified by Meyer et al. (2001): (1) Normal—(dE1): feces are hard but not hard, slightly deformed when dropped and placed on the floor; (2) Soft—(dE2): feces unformed, piled up, and scattered when falling; (3) Liquid—(dE3): feces dispersed in 6 mm deep flakes; (4) Water—dE4: with liquid consistency of stool.

(1) $$\mathrm{FCI}=\frac{[\left(\mathrm{dE}1\times 1\right)+\left(\mathrm{dE}2\times 2\right)+\left(\mathrm{dE}3\times 3\right)+\left(\mathrm{dE}4\times 4\right)}{\mathrm{Td}\;\times 4}\times 100$$

where dE1, dE2, dE3, and dE4 are the days with stool consistency scores = 1, 2, 3, and 4, respectively, and Td is the number of days of the trial period, 0–4 weeks and 4–8 weeks, respectively (Td = 35).

At the end of the experiment, the fecal samples were collected from 24 randomly selected goat kids so that there were 6 samples per treatment. Before collecting the feces of goat kids, the anus was cleaned three times with wipes and 75% alcohol. After that, about 1 g of fecal samples were collected with sterilized plastic gloves and placed in a 50 mL sterilized centrifuge tube for refrigeration in an ice bath. After the sample was diluted 10 times with buffered peptone water, the contents were shaken for 2 min, and the supernatant was taken. After the supernatant was serially diluted, 0.1 mL was applied to Violet red bile lactose agar (VRBLA, HIMEDIA^®^, Mumbai, MH, India) and TSA (HIMEDIA^®^, Mumbai, MH, India), then placed in a 37 °C incubator for 24 h to calculate Colony numbers (CFU/g feces) of Coliforms and *Bacillus*-likes bacteria.

### 2.7. Isolation of Peripheral Blood Mononuclear Cells and Granulocytes

In trial 1, the jugular blood collected in the EDTA tube was centrifuged at 400× *g* for 10 min at 4 °C, and the plasma was then removed for the later assays. The cell portion was diluted with RPMI-1640 (1:2) and layered onto Ficoll (Histopaque-1077, Sigma-Aldrich, St. Louis, MO, USA) for the density gradient separation. After 30 min, the samples were centrifuged at 450× *g* at room temperature, and the peripheral blood mononuclear cells (PBMCs) were collected. After a wash with cold phosphate-buffered saline (PBS), the cells were subjected to a live/dead count by a trypan blue exclusion method with a hemocytometer under a microscope as described elsewhere. After removal of the PBMCs from the samples, the red blood cell portions were subsequently lysed by commercial red blood cell lysis buffer (BioLegend, San Diego, CA, USA) to remove red blood cells and harvest granulocytes with centrifugation. Live PBMCs were used for lymphoblastogenesis assays. Purified granulocytes were then counted and subjected to a phagocytosis assay and an oxidative burst measurement.

### 2.8. Phagocytosis of Granulocyte

In trial 1, granulocytes of 1 × 10^6^ each were preseeded in a 96-well plate and then cocultured with fluorescently labeled bacteria at 1 × 10^7^ DioC18-labeled *Escherichia coli* (ATCC 25922) in a PBS solution at 37 °C for 90 min. By the end of the coincubation, 100 μL of trypan blue (1.25 mg/mL) was added to quench the residual DioC18-labeled *E. coli*. Phagocytosis of the granulocytes was determined using flow cytometry (Becton Dickinson FACSCaliburTM, Franklin Lakes, NJ, USA).

### 2.9. Oxidative Burst Measurement

In trial 1, the oxidative burst measurement followed the description of Ciapetti et al. [17], the granulocytes were co-incubated with unlabeled *E. coli* in a 37 °C incubator for 90 min, and the intracellular reactive oxygen species (ROS) was determined by adding 2′,7′-dichlorofluorescin-diacetate (DCF-DA). The generated DCF-DA was directly proportional to ROS as the process of the oxidative burst of the granulocytes was measured using flow cytometer.

### 2.10. Lymphoblastogenesis

In trial 1, isolated live PBMCs were diluted in 1 × 10^6^/mL and seeded onto a 96-well plate. Specific mitogens, all purchased from Sigma, USA, including 25 μg/mL phytohaemagglutinin (PHA), 20 μg/mL lipopolysaccharide (LPS), or 50 ng/mL phorbol 12-myristate 13-acetate plus 250 ng/mL ionomycin, were added to stimulate specific lymphocyte proliferation. Alamar Blue (Serotec Co., Oxford, UK) was added in the last 24 h of an entire 72 h culture at 37 °C in a 5% CO2 humidified incubator. The changes in specific fluorescence were measured using a microplate reader (FLX800, Bio-Tek Instruments, Inc., Winooski, VT, USA).

### 2.11. Skin-Swelling Measurement

In trial 1, eight goat kids from each treatment group were selected, 0.05 mL of PHA was injected into the skin of the goat kids’ left ear, and 0.05 mL of normal saline was injected into the skin of the right ear of the goat kids as a control group. After 12 h, the selected sample of eight goat kids was measured for the degree of swelling of the wattle with a digital thickness gauge.

### 2.12. Blood Immunoglobulin Level

In trial 1, the jugular blood was collected in an EDTA vacutainer tube and then centrifuged at 10,000× *g* for 2 min after clotting and stored individually at −80 °C. To determine the IgG, IgM, and IgA concentration, a commercial goat ELISA kit (Bethyl Laboratories, Montgomery, TX, USA) was used for 10 wks.

### 2.13. Hematological Traits

In trial 1, blood samples were obtained from the jugular vein with an EDTA vacutainer and stored at 4 °C until ready for analysis. The blood profiles (red blood cell (RBC), white blood cell (WBC), haemoglobin (Hgb), hematocrite (Hct), mean corpuscular volume (MCV), mean corpuscular hemoglobin (MCH), mean corpuscular hemoglobin concentration (MCHC), platelet (PLT), Leukocyte, Neutrophil extracellular traps (Net-s), lymphocytes (Lym-L), monocytes (Mono), eosinophils (Eos), and basophils (Baso) were estimated using automatic blood analyzer (ADVIA 120, Bayer, NY, USA). Serum haptoglobin was determined, using an enzyme-linked immunosorbent assay kit (TP801; Tri-Delta Diagnostics, Morris Plains, NJ, USA). For hematology, approximately 3 mL of blood was collected in tubes containing K2EDTA (BD Vacutainer, Plymouth, PL6 7BP, UK) and was analyzed immediately after collection. Blood samples for haptoglobin determinations were placed in serum separator tubes (BD Vacutainer) and were centrifuged at 3000× *g* for 15 min, and stored at −80 °C until assayed.

### 2.14. Clinical Blood Biochemistry

In trial 1, at the end of the experiment, blood samples were taken from the jugular vein of goat kids. After centrifuging (1700× *g*, 15 min), serum was stored at −40 °C for further analysis. The blood biochemistry of serum, including the activities of aspartate aminotransferase (AST), alanine aminotransferase (ALT), γ-glutamyl transpeptidase (γ-GT), lactate dehydrogenase (LDH), creatine kinase (CK), alkaline phosphatase (ALP), acid phosphatase (Acid-P), and the concentrations of calcium (Ca), phosphate (P), glucose, cholesterol, triglyceride (TG), total protein (TP), albumin (Alb), globulin (Glo), Alb/Glo (A/G), blood urea nitrogen (BUN), and creatinine were analyzed using an automatic blood chemical analyzer with Roche testing kits (Roche COBAS MIRA PLUS, Switzerland).

### 2.15. Apparent Digestibility

In trial 2, sixteen goat kids with similar body weights and good health were selected after trial 1 and used in this digestion trial beginning from 12-wk-old. The treatments were the same as in trial 1. Each treatment had four replications. The feed adaptation period was 3 d. Feed mix with 0.3% Cr_2_O_3_ was then fed to the goat kids for 4 d to evaluate the DM, CP, NDF, ADF, and total digestible nutrients (TDN). After collection of feces, feed and excreta samples were analyzed for DM, and nitrogen (N) according to AOAC [13]. Digestibility was calculated according to Williams et al. [18] as = 1 − [(Nf × Cd)/(Nd × Cf)] × 100, where Nf = nutrient concentration in excreta (% DM), Nd = concentration nutrient composition in the diet, Cd = concentration of chromium in the diet, Cf = concentration of chromium in the excreta.

### 2.16. Statistical Analysis

The data were analyzed using the GLM procedure [19], and the groups were compared using a one-way ANOVA with a Tukey post hoc test, where *p* < 0.05 indicated a statistically significant difference. The GHS and FCI in the general health performance were analyzed using the NPAR1WAY procedure [19]. The groups were compared using SAS^®^ macro implementation of a multiple comparison test according to Elliott and Hynan [20], where *p* < 0.05 indicated a statistically significant difference.

## 3. Results

### 3.1. The Physiochemical Characterizations of FFMSP

Table 2 shows the physicochemical characterizations of the FFMSP. The pH value and the counts of the Bacillus-like bacteria in the FFSMP were significantly increased after fermentation and decreased after drying but were still higher than those before fermentation (*p* < 0.05). After fermentation, the FFSMP can produce γ-PGA, acidic protease, neutral protease, alkaline protease, keratinase, surfactin, and other active ingredients.

### 3.2. Nutrient Composition and Amino Acid Composition of FFSMP

Table 3 shows the nutrient composition and amino acid composition of FFMSP. After fermentation, the total amino acid, total essential amino acid, histidine, lysine, and alanine of FFSMP were significantly higher than those of FSM (*p* < 0.05).

### 3.3. Growth Performance

Table 4 shows the effects of FFMSP on the growth performance of goat kids in the starter. The body weight, WG, and FCR of the FSM group were all inferior to those of the other groups (*p* < 0.05) during 5–10 weeks (7–12 weeks of age). The FFSMP groups showed an improvement in WG and FCR compared with the FSM group (*p* < 0.05), and the fish meal group was between them (*p* > 0.05) during 0–10 weeks.

### 3.4. Health Performance

Table 5 shows the effects of FFMSP on the health performance of goat kids in the starter diet. The fecal consistency index could improve by replacing the fish meal with the FFSMP as compared with the FSM group (*p* < 0.05), where the other groups were between them (*p* > 0.05) during 0 to 5 weeks of the experiment. The fecal consistency index of the FSM group was inferior to the group that replaced 100% of the fish meal with the FFSMP (*p* < 0.05) during 5–10 weeks of the experiment. Coliforms and *Bacillus*-like bacteria counts were improved by replacing 100% of the fish meal with the FFSMP group compared with the FSM group (*p* < 0.10).

### 3.5. Immunity

Table 6 shows the effects of the FFMSP on the immunity of goat kids in the starter diet. The oxygen burst in the group that replaced 100% of the fish meal with FFSMP was significantly higher than that in the FSM group (*p* < 0.05). The swelling reaction in the group with FFSMP replacing 50% of the fish meal was significantly higher than that in the FSM group (*p* < 0.05).

### 3.6. Hematological Traits

Dietary supplementation of FFSMP did not significantly affect the hematological traits (*p* > 0.05) of the goat kids, including RBC, WBC, Hgb, Hct, MCV, MCH, MCHC, PLT, Net-s, Lym-L, Mono, Eos, and Baso (not listed).

### 3.7. Clinical Blood Biochemistry

Table 7 shows the effects of the FFMSP on the clinical blood biochemistry of goat kids in the starter diet. The group that replaced 100% of the fish meal with FFSMP showed significantly higher values than those in the FSM group (*p* < 0.05) in the blood ALP activity, Glo, and P concentrations. The FSM group showed significantly lower values than those of each treatment group (*p* < 0.05) for the blood concentrations of TP and BUN.

### 3.8. Apparent Digestibility

Table 8 shows the effects of the FFMSP on the apparent digestibility of goat kids on the starter diet. The CP digestibility of the FSM group was significantly inferior to that of other groups (*p* < 0.05).

## 4. Discussion

Feather is a waste produced by the poultry industry, and its low protein bioavailability is due to its high keratin content. Hydrolyzing feathers using keratinase-producing microbial fermentation is an economical and environmentally friendly method for sustainable recycling in the animal feed industry, especially with the current high rise in feed costs [21,22]. After fermentation with CU33, the pH value and the bacterial count of the test matrix increased due to the decomposition and utilization of the matrix protein by microorganisms, so that the activities of protease and keratinase could be detected in the matrix. It shows that using the mixed matrix of feather meal and soybean meal is suitable for the nutritional metabolism of CU33. *Bacillus* strains can form endospores and have a certain degree of resistance to harsh external environments [23]. In this study, after drying FFSMP at 55 °C, the bacterial count of CU33 was still 7.54 log CFU/g, showing its tolerance to the drying environment. In previous studies, using *Bacillus* strains for fermentation, the counts of *Bacillus* could still maintain a certain number after drying, which is consistent with the results of this study [8,14,24].

γ-PGA is a polymer formed by the dehydration and condensation of the α-amino and α-carboxyl groups of the two glutamic acid molecules by *Bacillus* strains and is sticky. These properties improve animals’ abilities to utilize calcium salts, hence reducing the waste of dietary minerals (Chen et al., 2012). The excessive high viscosity of the fermentation product as a high content of γ-PGA, however, increases the drying time due to the low bulkiness of the substrate. Nie et al. (2015) found that 70% moisture had the highest γ-PGA yield as compared to 50–70% moisture in a fermented soybean meal and a rice husk solid culture medium. Our previous study also found that with the increased moisture of solid-state fermentation, the content and viscosity of γ-PGA increased (not listed). The current study uses 50% moisture for fermentation. The final γ-PGA content and viscosity of FFSMP were 1.8% and 1.67, respectively, which help product drying, subsequent feed processing and utilization.

Feather meal and soybean meal are fermented to degrade the proteins and improve the amino acid composition [2,25]. In this study, fermentation changed the contents of the total amino acid, total essential amino acid, histidine, lysine, and alanine, and the CU33 changed the amino acid composition after 2 days of aerobic fermentation. In this experiment, the general components of FFSMP including the moisture, total energy, crude protein, and the content of calcium and phosphorus did not change after 2 days of aerobic fermentation (*p* > 0.05). Since water participates in microbial biomass development and metabolic reactions, enzymatic activities, and transport of nutrients, extracellular metabolites, and gases during solid-state fermentation, the moisture required for fermentation with different substrates is about 30% to 85% [26]. In this study, the moisture was 50% during fermentation, and it did not over-ferment and evaporate, so its general composition did not change. Essential amino acids are nutrient sources that cannot be synthesized and are required to be supplemented in the animal body. FFSMP contains functional components such as probiotics, γ-PGA, protease, and surfactin, which help enhance the nutritive value of FFSMP.

Dietary treatment did not show a significant difference in growth performance between the groups (*p* > 0.05) during 0–5 weeks of the experimental period since milk replacer still accounted for 43–48% of the whole diet. The FFSMP groups showed a better WG and FCR than those of the FSM group (*p* < 0.05) during weeks 5–10 of the experiment (post-weaning) on account of the FFSMP in the milk replacer being increased to account for up to 70–72% of the dietary intake. Only the FFSMP groups were significantly better in the WG and FCR than the FSM group (*p* < 0.05) during weeks 0–10 of the experiment, while the fish meal group was between them (*p* > 0.05), indicating that FFSMP may contain ingredients that can promote growth and improve FCR since there were over 10^7^ CFU/g counts of *Bacillus*-like bacteria, as shown in Table 1, and FFSMP also contained other functional components such as γ-PGA that can promote the utilization of calcium salts, the protease that helped the utilization of proteins, and surfactin that is antibacterial. It appears that replacing 50% or 100% of the fish meal completely by adding 1% or 2% FFSMP to the trough feed can improve the growth performance of goat kids.

A lower GHS means that animals may face diarrhea, respiratory diseases, other infectious diseases, and high mortality in feeding [16]. In this study, dietary treatment did not show a significant difference in GHS among the groups (*p* > 0.05) due to the large variance among groups. Only the 100% fish meal replacement with the FFSMP showed a significant improvement in the fecal consistency index compared with the FSM group (*p* < 0.05) during weeks 0–5 and 5–10. While the dietary treatment did not show significant differences in WG and FCR among the kid groups during weeks 0–5, there was a significant improvement among the groups with the exception of the FSM group (*p* < 0.05) during weeks 5–10, indicating that using FFSMP in the starter diet could improve the growth performance and the fecal index of goat kids. Among them, replacing 100% of the fish meal with FFSMP outperformed other groups with the goat kids. The group that replaced 100% of the fish meal with FFSMP tended to increase the counts of *Bacillus*-like bacteria and decrease the counts of Coliforms bacteria in the feces compared with the FSM group (*p* < 0.10). *Bacillus* spp. can produce spores, resisting the pH and digestive enzymes of the stomach and intestinal environment, while feeding *Bacillus* spp. has an antagonistic effect on intestinal Coliforms. At the same time, the counts of Coliforms in feces will decrease with increasing dietary supplementation of *Bacillus* strains [27]. CU33 is a *Bacillus* strain that can produce spores. Therefore, as the supplementation of FFSMP increased in the diet, the counts of *Bacillus*-like bacteria increased in the feces. Meanwhile, CU33 resides in the digestive tract and could secrete surfactin, which has a bacteriostatic effect on reducing Coliform counts in feces.

With the inflammatory response, polymorphonuclear leukocytes in phagocytes play an essential role. Leukocytes reach the peripheral blood vessels of the inflamed site by chemotaxis and kill bacteria by phagocytosis and oxygen bursts [28]. In this study, the oxygen burst effect of the FFSMP group that replaced 100% of the fish meal group showed significantly higher values than that of the FSM group (*p* < 0.05). This means that replacing 100% of the fish meal with FFSMP can increase the oxygen burst effect of goat kids’ polymorphonuclear leukocytes on *Escherichia coli* to increase the killing effect of leukocytes on foreign invading bacteria. The skin swelling test is a rapid, low-cost immunological method to directly assess an individual’s pro-inflammatory capacity on the farm [29]. Subcutaneous injection of the lectin PHA allows immune cells to infiltrate the inoculation site, resulting in swelling of the subcutaneous tissue [30]. In this study, the 50% replaced fish meal with FFSMP group showed a significantly higher value for the swelling reaction of PHA than that in the FSM group (*p* < 0.05), while the rest of the groups were between the two (*p* > 0.05). This indicated that dietary supplementation of fishmeal or FFSMP could help stimulate the immune response of the goat kids, which was beneficial in alleviating environmental stress impact on diarrhea, thereby improving growth.

There were no differences in hematological traits among the groups (*p* > 0.05), indicating that feeding FFSMP had no adverse effect on the physiological blood function of the goat kids. Generally, young animals are in a rapid growth stage and the decomposition of osteoclasts in the bones results in higher ALP activity and P concentration in their blood [31]. This is consistent with the results of using 2% FFSMP to make the blood of goat kids have higher ALP activity and P concentration, which is also reflected in the higher body weight and WG of the 2% FFSMP group than the control group. Glo is the value obtained by subtracting Alb from serum TP. However, blood Glo is susceptible to an increase or decrease in Alb in serum. It is not easy to determine its significance from determining total protein content, so A/G is usually used as the basis for determination [31]. The FFSMP group showed a higher concentration of Glo (*p* < 0.05), but there was no significant difference in Alb and A/G among the groups (*p* < 0.05), indicating that FFSMP had no adverse effect on protein transport and metabolism in the goat kids (*p* < 0.05).

Blood urea nitrogen is a protein metabolite, and its concentration is affected by the renal excretion efficiency and the rate of body synthesis. Factors such as protein-rich diets or diseases increase its concentration. In this iso-nitrogenic diet study, increased BUN of the treatment group reflected an increase in the protein digestibility of the diet by the goat kids (Table 8). Since the high-quality fish meal is rich in protein and has a digestibility of up to 90% [32], it is often used as a feed for ruminant cubs to replace part of the milk powder used. The matrix macromolecular proteins of FFSMP were converted into small molecular peptides or amino acids after the microorganisms were fermented so that the proteins or amino acids could be better digested and absorbed [2,14]. Dietary supplementation of 1–2% FFSMP could promote WG of the 5–10-week-old goats compared with the control group, improve the FCR performance (*p* < 0.05), and achieve a compatible performance with the fish meal group (Table 4). In addition, an increase in FFSMP also increased the blood TP content compared with the control group (*p* < 0.05), which reflected that the FFSMP has positive effects on protein digestion, metabolism, and physiology of goat kids.

Alanine aminotransferase is a liver-specific enzyme. When the serum ALT activity increases, there may be liver disease. AST is widely distributed in various tissues, and liver cell injury, muscle injury, and myocardial necrosis can increase serum AST activity. LDH is widely distributed in various tissues and can be used to indicate tissue damage [31]. In this experiment, dietary treatment did not show any significant difference in ALT, AST, and LDH among the groups (*p* > 0.05), indicating that the addition of FFSMP had no adverse effects on the goat kids’ tissues, organs, and physiology.

The digestibility of DM, GE, and TDN in goat kids showed no significant difference among each treatment group (*p* > 0.05). However, the digestibility of CP in FSM was significantly lower than that in other groups (*p* < 0.05). The protein of high-quality fish meal is relatively easy to digest, while feather meal contains 90% keratin [33,34,35], which is highly resistant to various proteolytic microorganisms, enzymes, and chemical and physical damage [36], and thus is not easily digested and utilized. The FFSMP used in this trial effectively degraded the keratin from the raw feathers by inoculating CU33, the screened feather-decomposing bacteria. Using microbial fermentation technology, the macromolecules of the FFSMP were converted into small molecules, and combined with the probiotics and unknown growth factors, so that FFSMP improved the apparent digestibility of CP and showed an improving effect on growth performance, diarrhea status, and health performance of goat kids. Moreover, FFSMP can completely replace high-quality fish meal in the starter diet.

## 5. Conclusions

Dietary supplementation with 2% FFSMP in the diet can promote the growth of goat kids by improving CP digestibility, diarrhea status, and immunity, and can, therefore, completely replace the fish meal as a starter diet.

## Figures and Tables

**Table 1:** Ingredient composition and nutrient level of starter (DM basis).

Ingredient	FSM ^1^	FFSMP ^2^ Replace Fish Meal, %		
		0	50	100
Alfafa meal	15	15	15	15
Yellow corn, grain	54.38	55.60	54.94	54.38
Sugar cane molasses	3	3	3	3
Soybean meal, CP 43%	19.3	21.5	20.3	19.3
Full-fat soybean meal, CP 38%	2.75	0	1.5	2.75
Fish meal, CP 65%	0	2	1	0
FFSMP, CP 63.8%	0	0	1	2
FSM, CP 63.8%	2	0	0	0
Salts	0.8	0.7	0.75	0.8
Dicalcium phosphate	1.15	0.7	0.95	1.15
Limestone, pulverized	1.32	1.2	1.26	1.32
Vitamin premix ^3^	0.2	0.2	0.2	0.2
Mineral premix ^4^	0.1	0.1	0.1	0.1
Total	100	100	100	100
Calculated value				
CP, %	17.2	17.2	17.2	17.2
ME, Kcal/kg	2886	2886	2886	2886
NDF, %	13.9	14.3	14.0	13.9
ADF, %	7.96	8.13	8.03	7.96
Calcium, %	0.99	0.99	0.99	0.99
Phosphorus, %	0.51	0.51	0.51	0.51
Analyzed value				
CP, %	17.7	17.4	17.8	17.5
NDF, %	14.2	14.9	14.6	14.4
ADF, %	7.86	8.21	8.11	7.98
Calcium, %	1.05	1.04	1.09	1.11
Total phosphorus, %	0.56	0.55	0.53	0.57

^1^ FSM = Feather–soybean meal. ^2^ FFSMP = Fermentation feather–soybean meal product inoculated with *Bacillus amyloliquefaciens* CU33. ^3^ Vitamin premix supplied per kilogram of diet: vitamin A, 1,000,000 IU; vitamin D_3_, 270,000 IU; vitamin E, 2900 IU. ^4^ Mineral premix supplied per kilogram of diet: Cu, 5000 mg; Fe, 9000 mg, Zn, 8000 mg, Mn, 6000 mg; Co (CoCO_3_, 49.5% Co) and Se (Na_2_SeO_3_, 45.7% Se), 0.25 mg.

**Table 2:** Physical and chemical analysis of FFSMP ^1^.

Items	FFSMP
pH value	
Initial	6.24 ± 0.01 ^z^
Fermentation	8.18 ± 0.02 ^x^
Dry	6.53 ± 0.01 ^y^
SEM	0.006
*p*-value	0.001
*Bacillus* like, log CFU/g	
Initial	6.71 ± 0.01 ^z^
Fermentation	8.32 ± 0.06 ^x^
Dry	7.54 ± 0.02 ^y^
SEM	0.019
*p*-value	0.001
Functional ingredients of dry product	
γ-PGA, %	1.80 ± 0.09
Viscosity, score	1.67 ± 0.08
Neutral protease, u/g	426 ± 5.79
Alkaline protease, u/g	357 ± 19.8
Acid protease, u/g	466 ± 17.4
Keratinase, u/g	411 ± 8.79
Surfactin, mg/g	1.13 ± 0.08

Data are the means ± SD of 4 batches of each fermented product, n = 4. ^x–z^ Means in the same column with different superscripts are significantly different (*p* < 0.05). ^1^ FFSMP = Fermented feather–soybean meal product.

**Table 3:** Amino acid composition analysis of the FFSMP.

Items	FSM ^1^	FFSMP ^2^	SEM	*p*-Value
Chemical analysis				
Moisture, %	10.6	10.6	0.05	0.71
CP, %	63.8	63.9	0.18	0.86
Gross energy, kcal/kg	3055	3046	5.38	0.32
Ca, %	0.24	0.24	0.01	0.57
TP, %	0.54	0.56	0.012	0.23
Amino acid analysis				
Total amino acid, %	63.9 ^b^	64.3 ^a^	0.08	0.02
Totla essential amino acid, %	27.1 ^b^	27.5 ^a^	0.08	0.01
Arg	4.29	4.32	0.02	0.44
His	0.81 ^b^	0.86 ^a^	0.01	0.002
Ile	3.10	3.16	0.03	0.22
Leu	5.76	5.80	0.05	0.56
Lys	2.20 ^b^	2.35 ^a^	0.02	0.003
Met	0.63	0.68	0.02	0.05
Phe	3.15	3.18	0.03	0.56
Thr	2.85	2.75	0.03	0.06
Val	4.32	4.44	0.04	0.06
Total nonessential amino acid, %	36.8	36.8	0.10	0.80
Cys	1.86	1.89	0.03	0.60
Asp	5.41	5.47	0.04	0.34
Ser	6.18	6.21	0.04	0.61
Glu	8.90	8.96	0.04	0.27
Pro	4.91 ^a^	4.77 ^b^	0.02	0.001
Ala	4.50 ^b^	4.60 ^a^	0.03	0.04
Gly	3.08 ^a^	2.93 ^b^	0.03	0.01
Tyr	1.93	1.99	0.03	0.22

Data are the means of 3 batches of each fermented product, n = 3. ^a, b^ Means in the same row with different superscripts are significantly different (*p* < 0.05). ^1^ FSM = Feather–soybean meal. ^2^ FFSMP = Fermentation feather–soybean meal product inoculated with *Bacillus amyloliquefaciens* CU33.

**Table 4:** Effects of dietary addition of FFSMP on growth performances of goat kids.

Exp. Period	FSM ^1^	FFSMP ^2^ Replace Fish Meal, %			SEM	*p*-Value
		0	50	100		
	Body weight, kg					
Initial	4.42	4.37	4.23	4.37	0.22	0.93
5 wk	6.86	6.89	6.72	7.14	0.34	0.86
10 wk	10.35 ^b^	12.02 ^a^	12.11 ^a^	12.24 ^a^	0.50	0.04
	Total feed intake ^3^, kg					
0–5 wks	5.62	6.15	5.56	5.82	0.23	0.29
5–10 wks	16.23	17.57	16.17	16.79	0.69	0.46
0–10 wks	21.84	23.72	21.73	22.61	0.75	0.24
	Starter intake, kg					
0–5 wks	2.94	3.49	2.85	3.12	0.23	0.23
5–10 wks	11.30	12.64	11.25	11.87	0.67	0.44
0–10 wks	14.24	16.13	14.10	14.99	0.73	0.20
	Weight gain, kg					
0–5 wks	2.44	2.52	2.49	2.77	0.25	0.80
5–10 wks	3.49 ^b^	5.13 ^a^	5.39 ^a^	5.10 ^a^	0.33	0.001
0–10 wks	5.93 ^b^	7.65 ^ab^	7.88 ^a^	7.87 ^a^	0.45	0.01
	Feed conversion ratio (feed intake/weight gain)					
0–5 wks	2.30	2.44	2.22	2.10	0.29	0.92
5–10 wks	4.65 ^a^	3.42 ^b^	3.00 ^b^	3.29 ^b^	0.28	0.001
0–10 wks	3.68 ^a^	3.10 ^ab^	2.76 ^b^	2.87 ^b^	0.19	0.01

Data are the means of 8 pens of goat kids, n = 8. ^a, b^ Means in the same row with different superscript differ significantly (*p* < 0.05). ^1^ FSM = Feather–soybean meal. ^2^ FFSMP = Fermentation feather–soybean meal product inoculated with *Bacillus amyloliquefaciens* CU33. ^3^ Total feed intake = milk replace + starter.

**Table 5:** Effects of dietary addition of FFSMP on health performance of goat kids.

Periods	FSM ^1^	FFSMP ^2^ Replace Fish Meal, %			SEM	*p*-Value
		0	50	100		
	General health score **					
0–5 wks	10.7	17.0	18.9	19.4	-	0.19
5–10 wks	12.1	14.6	19.4	20.0	-	0.22
0–10 wks	10.8	16.0	19.4	19.8	-	0.18
	Fecal consistency index, % **					
0–5 wks	25.0 ^a^	15.4 ^ab^	13.7 ^ab^	11.9 ^b^	-	0.02
5–10 wks	25.1 ^a^	14.1 ^ab^	14.6 ^ab^	12.2 ^b^	-	0.03
	Fecal bacterial ***					
Coliforms	5.73	4.71	4.75	3.84 ^*^	0.49	0.09
*Bacillus*-likes	3.46	3.65	4.71	4.77 ^*^	0.42	0.07

^a, b^ Means in the same row with different superscript differ significantly (*p* < 0.05). * *p* < 0.1. ** Data are the means of 8 pens of goat kids, n = 8. *** Data are the means of 6 pens of goat kids, n = 6. ^1^ FSM = Feather–soybean meal. ^2^ FFSMP = Fermentation feather–soybean meal product inoculated with *Bacillus amyloliquefaciens* CU33.

**Table 6:** Effects of dietary addition of FFSMP on immune traits of goat kids.

IItems	FSM ^1^	FFSMP ^2^ Replace Fish Meal, %			SEM	*p*-Value
		0	50	100		
555 wks	Mean fluorescence intensity					
Phagocytosis	2.01	2.61	2.28	2.82	0.27	0.15
Oxygen burst	867 ^b^	902 ^ab^	1013 ^ab^	1088 ^a^	53.7	0.03
	Specific fluorescence					
Phytohaemagglutinin	11,923	13,269	10,642	9400	1108	0.10
Lipopolysaccharide	8858	7898	9474	10,841	965	0.20
PMA/ION ^3^	7309	8657	7284	7108	810	0.51
	Swelling degree, mm					
After injection of 0 h	8.66	10.11	9.43	9.71	0.72	0.55
After injection of 12 h	9.84	12.55	12.20	12.05	0.80	0.09
Swelling	1.18 ^b^	2.43 ^ab^	2.77 ^a^	2.34 ^ab^	0.38	0.03
10 wks	Immunoglobulin, mg/dL					
IgA	0.7	0.67	0.65	0.72	0.06	0.81
IgG	2.89	2.14	2.13	2.75	0.40	0.40
IgM	0.21	0.36	0.30	0.21	0.06	0.06

Data are the means of 8 pens of goat kids, n = 8. ^a, b^ Means in the same row with different superscript differ significantly (*p* < 0.05). ^1^ FSM = Feather–soybean meal. ^2^ FFSMP = Fermentation feather–soybean meal product inoculated with *Bacillus amyloliquefaciens* CU33. ^3^ PMA/ION: Phorbol-12-myristate-13-acetate/Ionomycin.

**Table 7:** Effects of dietary addition of FFSMP on clinical blood biochemistry of goat kids.

Items	FSM ^1^	FFSMP ^2^ Replace Fish Meal, %			SEM	*p*-Value
		0	50	100		
AST, U/L	346	401	399	368	22.0	0.25
ALT, U/L	44.0	42.9	42.8	46.3	2.0	0.74
γ-GT, U/L	24.3	27.8	25.9	28.6	1.30	0.10
LDH, U/L	3427	3345	3126	3328	0.12	0.60
CK, U/L	11,025	10,328	12,041	10,928	641	0.44
Alk-P, U/L	1258 ^b^	1735 ^ab^	1902 ^ab^	2029 ^a^	186	0.04
Acid-P, U/L	0.33	0.21	0.23	0.28	0.07	0.71
Ca, mg/dL	9.52	9.33	9.89	9.83	0.34	0.62
P, mg/dL	7.81 ^b^	8.26 ^ab^	8.5 ^ab^	8.63 ^a^	0.20	0.04
Glucose, mg/dL	63.6	68.9	66.4	68.5	3.10	0.62
Cholesterol, mg/dL	62.4	65.8	61.4	62.6	2.92	0.74
Tg, mg/dL	22.6	24.4	22.9	23.8	0.98	0.58
TP, g/dL	6.66 ^b^	7.50 ^a^	7.68 ^a^	7.79 ^a^	0.18	0.01
Alb, g/dL	2.33	2.55	2.61	2.65	0.12	0.23
Glo, g/dL	4.34 ^b^	4.95 ^ab^	5.06 ^a^	5.14 ^a^	0.17	0.01
A/G	0.53	0.53	0.52	0.52	0.04	1.00
BUN, mg/dL	13.5 ^b^	18.0 ^a^	20.4 ^a^	19.6 ^a^	0.98	0.01
Creatinine, mg/dL	1.03	1.01	1.05	1.01	0.08	0.98

Data are the means of 8 pens of goat kids, n = 8. Abbreviations: A/G, albumin/globulin; Acid-P, acid phosphatase; Alb, albumin; ALP, alkaline phosphatase; ALT, alanine aminotransferase; AST, aspartate aminotransferase; BUN, blood urea nitrogen; Ca, calcium; CK, creatine kinase; Glo, globulin; LDH, lactate dehydrogenase; P, phosphate; TG, triglyceride; TP, total protein; γ-GT, γ-glutamyl transpeptidase. ^a, b^ Means in the same row with different superscript differ significantly (*p* < 0.05). ^1^ FSM = Feather–soybean meal. ^2^ FFSMP = Fermentation feather–soybean meal product inoculated with *Bacillus amyloliquefaciens* CU33.

**Table 8:** Effects of starter addition of FFSMP on apparent digestibility of goat kids.

Item	FSM ^1^	FFSMP ^2^ Replace Fish Meal, %			SEM	*p*-Value
		0	50	100		
DM, %	71.3	72.9	71.8	72.2	0.66	0.41
CP, %	69.2 ^b^	73.2 ^a^	72.5 ^a^	73.9 ^a^	0.48	0.001
Gross energy, %	71.13	72.60	73.22	72.96	1.05	0.52
TDN, %	70.9	72.5	72.0	71.9	0.61	0.34

Data are the means of 4 pens of goat kids, n = 4. ^a, b^ Means in the same row with different superscript differ significantly (*p* < 0.05). ^1^ FSM = Feather–soybean meal. ^2^ FFSMP = Fermentation feather–soybean meal product inoculated with *Bacillus amyloliquefaciens* CU33.

## Data Availability

The data presented in this study are available upon request from the corresponding author.
